# Short-Term Reciprocity in Macaque’s Social Decision-Making

**DOI:** 10.3389/fnbeh.2019.00225

**Published:** 2019-09-24

**Authors:** Sébastien Ballesta, Gilles Reymond, Jean-René Duhamel

**Affiliations:** ^1^Centre de Neuroscience Cognitive, Centre National de la Recherche Scientifique, UMR 5229, Bron, France; ^2^Département de Biologie Humaine, Université Lyon 1, Villeurbanne, France

**Keywords:** reciprocation, social neuroscience, action understanding, imitation, prosocial behaviour, food-sharing, hostility management

## Abstract

Primates live in complex social environments, where individuals create meaningful networks by adapting their behavior according to past experiences with others. Although free-ranging primates do show signs of reciprocity, experiments in more controlled environments have mainly failed to reproduce such social dynamics. Hence, the cognitive and neural processes allowing monkeys to reciprocate during social exchanges remains elusive. Here, pairs of long-tailed macaques (*Macaca fascicularis*) took turns into a social decision task involving the delivery of positive (juice reward) or negative (airpuff) outcomes. By analyzing the contingencies of one partner’s past decisions on the other’s future decisions, we demonstrate the presence of reciprocity, but only for the exchange of negative outcomes. Importantly, to display this decisional bias, the monkey needs to witness its partner’s decisions, since non-social deliveries of the same outcome did not have such effect. Withholding of negative outcomes also predicted future social decisions, which suggest that the observed tit-for-tat strategy may not only be motivated by retaliation after receiving an airpuff but also by the gratefulness of not having received one. These results clarify the apparent dichotomy within the scientific literature of reciprocity in non-human primates and suggest that their social cognition comprise revenge and gratitude.

## Introduction

During exchanges of positive or negative actions between individuals, prior outcomes may influence further social interactions. Many studies show that free-ranging primates display reciprocity in a range of social behaviors but reciprocal behavior has rarely been observed for food exchanges (Packer, 1977; Seyfarth and Cheney, 1984; de Waal and Luttrell, 1988; Ventura et al., 2006; Schino, 2007; Schino and Pellegrini, 2009; Carne et al., 2011; Weinstein and Capitanio, 2012; Xia et al., 2013; Amici et al., 2014; Borgeaud and Bshary, 2015; Molesti and Majolo, 2015, 2017). Surprisingly, under experimentally controlled conditions, monkeys failed to display signs of reciprocity (Brosnan et al., 2009; Yamamoto and Tanaka, 2009; Pelé et al., 2010; Suchak and de Waal, 2012; Claidière et al., 2015). This apparent lack of valuation of prior exchanges might be interpreted in several ways, such as real absence of short-term reciprocity, specific insensitivity to food exchanges, or poor understanding of their own and/or other’s agency inside such experimental apparatus (Jaeggi et al., 2013; Drayton and Santos, 2014). Hence, previous experimental studies may have underestimated the presence of reciprocity in non-human primates (Schweinfurth and Call, 2019). Here, we assessed the presence of reciprocity in well-controlled social decisions between young male long-tailed macaques (*Macaca fascicularis*). We designed a task where two monkeys faced each other, and alternately made social decisions involving juice or airpuff delivery to the partner or to an empty space (called “nobody” hereafter; Ballesta and Duhamel, 2015). In this task, the actor receives the same amount of juice drops after each of its social decisions, whether or not it involves positive (juice) or negative (airpuff) outcomes for its partner. Thus, choice in social decisions only differed as whether these would affect the partner or not, while the benefit for self was unchanged regardless of the choice. Importantly, each experimental session includes control non-social decisions where the monkey had to choose between the delivery of an outcome to himself or to nobody. It should be noted that the delivery of an airpuff is a negative somatosensory experience which can be compared to other negative stimuli used in experiments on social exchanges in humans (Shergill et al., 2003). The interpretation of its significance by non-human primates may be more straightforward than that of a food (or juice) exchange, which is, for instance, affected by several other factors such as satiety, spatial proximity of peers, and social hierarchy (Watson and Caldwell, 2009).

## Materials and Methods

### Animals

Four non-kin juvenile male long-tailed macaques (*Macaca fascicularis*; aged 3 ± 0.15 years, weight 5.7 ± 0.8) were used as subjects. They were housed as a mini-colony in a relatively large enclosure (15 m^3^) allowing direct physical interactions but also isolation through a system of sliding partitions. When isolated, monkeys could communicate visually and vocally. Animals were fed with monkey chow, fresh fruits and vegetables. The monkeys were maintained under scheduled access to fluid in order to maintain optimal motivation for juice. Extra fluid and fruits could be given as needed at the end of each day to maintain the animals’ proper fluid balance. In addition, animals had at least 1 day of free access to water each week. Animals were weighted before each experimental session. The difference in weight of the animals between periods of *ad libitum* and restricted access to fluids was always inferior to the ethical limit of 10% [which is in the range of what non-human primates can experience in the wild (Zurovsky et al., 1984)]. According to recent scientific investigations, it is unlikely that such fluid restriction protocol had caused any physiological or psychological harm to the subjects (Gray et al., 2016). The cages were enriched with different toys, computer-based enrichment and substrates that promote social play, curiosity, object manipulation and foraging. This study was part of a larger project on social cognition involving neurophysiological and eye movement recordings which required specific surgical procedure to implant a head restraining device. Neural recordings were not performed in the considered dataset. Despite that this surgical procedure was not needed for this particular study, it does not impede the scientific validity of our results. In fact, in many other ethically accepted social neuroscience studies, non-human primates has already proven their ability to express coherent socio-cognitive behaviors (Chang et al., 2011; Yoshida et al., 2011; Haroush and Williams, 2015) that were consistent with naturally occurring social behaviors (Ballesta and Duhamel, 2015). Briefly, after a single sterile surgery performed under isoflurane anesthesia, the monkeys were then left to recover for at least 1 month with the proper antibiotic coverage, and pain-relievers were given as needed. All experimental procedures were approved by the animal care committee (Department of Veterinary Services, Health and Protection of Animals, permit number 69-029-0401) and the Biology Department of the University Claude Bernard Lyon 1, in conformity with the European Community standards for the care and use of laboratory animals (European Community Council Directive No. 86-609).

### Behavioral Procedures

**Figure 1:**
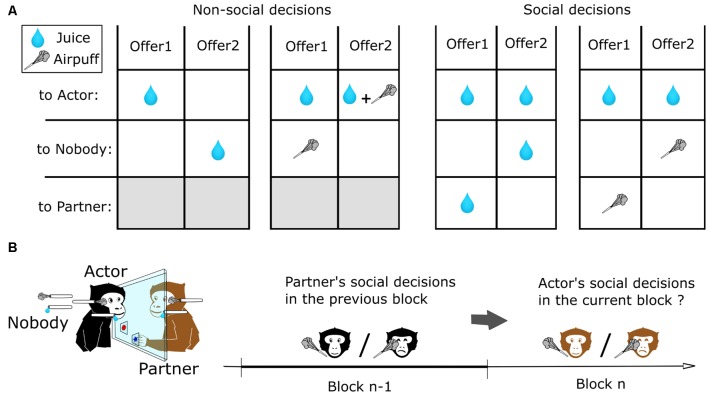
Experimental task and analysis to measure reciprocity during social exchanges. **(A)** Set of outcomes used for non-social and social decisions. Different offers configurations were presented on successive trials from a pre-defined set of Four possible types of decisions, in randomly interleaved order. Each cue shape was associated with a unique set of outcomes to the actor (self), the partner (other) or nobody. **(B)** Method used to analyze the effect of prior decisions on next decisions. The two monkeys alternated as actor and partner on successive blocks of 30 s. We compared the decisions of an actor monkey depending on the nature of the decisions of the other monkeys towards him on the preceding block of trials.

The setup was designed to allow neurophysiological and eye movement recordings while the two monkeys interacted visually with each other and made behavioral choices using the touch panel interface (Figure 1). Animal training and social decision task procedures are the same as in Ballesta and Duhamel (2015). Briefly, juice and airpuffs (four bars) were delivered using a gravity-based solenoid device (Crist Instruments, Hagerstown, MD, USA) and pressure gauge. Using a video projector and two semi-transparent mirrors (Beam splitter, 30% Reflection, 70% transmission, Edmund Optics Inc., Barrington, NJ, USA), the same visual stimuli were virtually projected in the visual plane of the two touch panels. Trials began with the appearance of a central colored square stimulus, which specified the actor monkey on that trial. When touched, this stimulus triggered the appearance of two distinct cues, each associated with a unique set of outcomes to the actor (self), the partner (other) or nobody (Figure 1A). After 500 ms, the square target was extinguished, and the monkey made its choice by touching the corresponding cue. After 1,500 ms, the outcomes were delivered. The two monkeys alternated as actor and partner on successive blocks of 30 s during which it performed an average of 3.14 (±0.9) trials Different choice configurations were presented on successive trials from a pre-defined set of four possible offers (Figure 1A), in randomly interleaved order. Consequently, when considering the order of presentation of the different types of offer, 558 different blocks of trials were performed by the subjects in this dataset. Unique sets of visual cues were associated with the outcomes delivered to each monkey. Behavioral control and visual displays were under the control of PCs running the REX/VEX system (Hays et al., 1982). All analog and digital data were logged and synchronized using Spike2 (Cambridge Electronic Design, Cambridge, UK).

### Data Analysis

Data from 49 experimental sessions (3,531 blocks of trials 14,356 decisions: 7,186 social and 7,170 non-social decisions) where the actor and the partner monkey both performed more than 60 trials were analyzed using custom scripts written in Matlab R2015a (The Mathworks). Average working duration of the subjects was 55 min (±15). We compared the decisions of an actor monkey depending on the nature of the decisions of the other monkeys towards him on a preceding block of trials (Figure 1B). In order to categorize the level of prosociality of a block of trials, we computed a score for each preceding block by calculating the difference of each type of outcomes (for instance the difference in the number of airpuffs delivered to the partner or to nobody for each social decision of this kind). The computation of the previous block score (*pbs*) can be expressed by the following equation:

(1) $$pbs=\sum_{n=0}^{3}\delta_{n}$$

**Figure 2:**
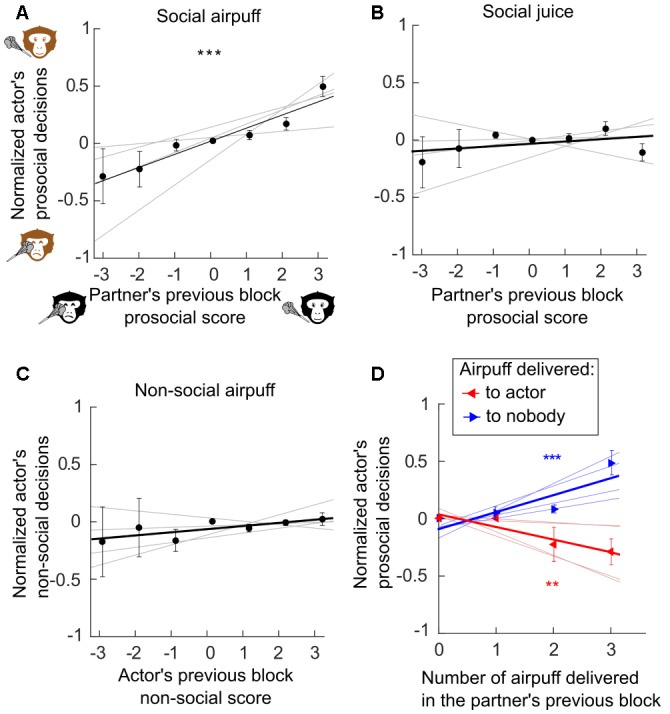
Previous delivery and avoidance of negative outcomes influenced subsequent social decisions. We assess whether prior decisions of a monkey (the partner or the actor) can influence the actor’s subsequent decisions. **(A)** Reciprocity between the social exchanges of airpuff (Generalized linear mixed effect models, GLME, *R*^2^ = 0.57, *F* = 34.6, *p* < 0.001). **(B)** Absence of reciprocity between the social exchanges of juice reward (GLME, *R*^2^ = 0.08, *F* = 1.63, *p* = 0.21). **(C)** Non-social delivery of airpuff does not influence social decision involving airpuff (GLME, *R*^2^ = 0.04, *F* = 1.16, *p* = 0.29). **(D)** Previous delivery and avoidance of airpuff predicted subsequent social decisions. (Number of airpuff delivered to nobody, GLME, *R*^2^ = 0.57, *F* = 21.3, *p* < 0.001; Number of airpuff delivered to the partner, GLME, *R*^2^ = 0.46, *F* = 10.3, *p* = 0.006). **(A–D)** Actors mean decision rates were normalized so that positive or negative value indicates, respectively, an increase or decrease of the prosocial decisional tendencies of the actor (see “Materials and Methods” section). Error bars represent standard error of the mean (SEM). Gray lines represent individual’s regressions. ***p < 0.001.

With δ being the outcome of a decision in each trial of the previous block and *n* the number of time that this type of decision was proposed in the previous block. Note that δ was equal to +1 and −1 when a prosocial or antisocial outcome was chosen, respectively (Figures 2A,B). To control for the non-social mere effect of receiving an airpuff, the same analysis was performed using the number of airpuff delivered to the actor by the actor himself in preceding block (Figure 2C). A similar score was computed when considering the number of each outcome delivered in the preceding block (Figure 2D). Then, for a given type of decision, actor’s mean decision rates (*dr*) were calculated as follow for each previous block score:

(2) $$dr_{x}=\frac{{\left(\frac{\#\text{of prosocial outcomes}}{\text{total \# of outcomes}}\right)}_{pbs=x}}{{\left(\frac{\#\text{of prosocial outcome}}{\text{total \# of outcome}}\right)}_{pbs=0}}-1$$

*x* is the value of the previous block score (pbs, used to determine if during the previous block the social decisions were neutral, pro- or antisocial). As the decision type was drawn randomly on each trial, each block contained between 0 and 4 decisions of each type. Hence, *x* could vary between −4 and 4 but was constrained between −3 and 3 as extremes data points were present in less than 1% of the blocks. Note that a score equal to zero also includes blocks where this type of decision was absent from the previous block. A positive or negative value of *dr* indicates, respectively, an increase or decrease of the prosocial decisional tendencies of the actor. Linear fittings were performed in Matlab using the function *fitglme* and always considered actors’ identity as a random factor.

## Results

**Figure 3:**
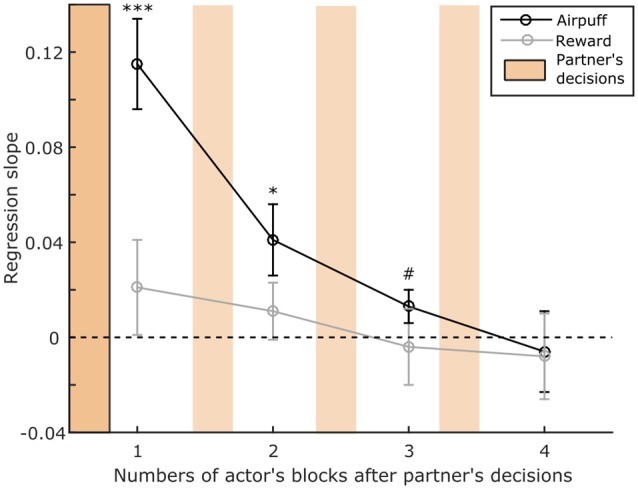
Persistence of the influence of partner’s social decisions on subsequent actor’s social decisions. We assess whether prior decisions of the partner monkey can influence the actor’s subsequent decisions in timescale longer than two consecutive blocks. The observed reciprocity when partner’s social decisions involved aipuff (*p* < 0.001) persists until the second actor’s block (*p* = 0.012). A non-significant trend can be observed on the third actor’s block (*p* = 0.056) but not on the fourth one (*p* = 0.74). We did not find any effect of partner’s social decisions involving rewards on same type of next actor’s social decisions (all *p* > 0.05). Y axis represents the slope of the regression (Beta coefficient of the GLME). X axis represents the number of subsequent actor’s blocks. Note that the two monkeys alternated as actor and partner on successive blocks of 30 s during which it performed an average of 3.14 (±0.9) trials. Error bars represent SE. *p < 0.05; ***p < 0.001; ^#^*p* < 0.1.

The results were analyzed for all the possible pairs composed by four young male long-tailed macaques, who switched roles as actor and partner every 30 s during a social decision task (Figure 1). The apparatus and the task were completely symmetric allowing considering the behavior of both of the animals during the 49 sessions, which represent 14,356 decisions. The decisions of the subjects were on average rational, for decision concerning self only, and prosocial, for decisions implicating the partner. The monkeys preferred to grant and avoid airpuffs (97% ± 2, 90% ± 3, Wilcoxon signed-rank test *p* < 0.01) to self, and showed a similar, though less pronounced, tendency for the same outcomes to their partners (68% ± 3 and 60% ± 4 for juice and airpuff, respectively, Wilcoxon signed-rank test *p* < 0.01). In order to assess the presence of contingency within decisions of each subject, we measured the effect of its own decisions in the prior block (i.e., made within at most 60 s) onto its next decisions. In addition, to assess the presence of contingency between decisions of the two subjects, we measured the effect of the other monkey’s decisions in the prior block on the actor next decisions. Each previous block of partner’s social decisions was scored by computing the difference in the number of each outcome for a given type of decision (see “Materials and Methods” section). Generalized linear mixed effect models (GLME, see “Materials and Methods” section) were computed to test whether the partner’s previous decisions were a significant predictor of the actor’s subsequent decisions (Figure 2). This analysis revealed that the partner’s social decision involving airpuff delivery in the previous block was a significant predictor of the actor’s social decisions of the same type in the current block (Figure 2A, GLME, *R*^2^ = 0.57, *F* = 34.6 *p* < 0.001). However such relation was not found when the social decisions involved juice reward (Figure 2B, GLME, *R*^2^ = 0.04, *F* = 1.1, *p* = 0.31) or when the social decisions of the partner’s and actors were of different type (past social decision: airpuff, current social decision: reward, GLME, *R*^2^ = 0.08, *F* = 1.63, *p* = 0.21; past social decision: reward, current social decision: airpuff, GLME, *R*^2^ = 0.12, *F* = 3.8, *p* = 0.07). Importantly, to control for the mere effect of receiving an airpuff independently from the identity of the sender, we also compared the next social decisions of the actor according to its own prior non-social decisions (e.g., whether the actor had sent airpuff to its own face on a prior block), and did not find significant results (Figure 2C, GLME, *R*^2^ = 0.04, *F* = 1.16, *p* = 0.29). This result suggests that having recently received an airpuff (which could plausibly make the monkey irritable, irrational, or less attentive to the partner’s fate) is not by itself sufficient to increase the delivery of this aversive outcome to the partner. In order to test if the measured effect persisted over several blocks, timescales longer than two consecutive blocks were considered (Figure 3). We found that the observed reciprocity when social decisions involved aipuff persisted until the second actor’s block (*p* = 0.012) and a non-significant trend can be observed for the third actor’s block (*p* = 0.056) but not until the fourth one (*p* > 0.05). Again, we did not find any effect of partner’s social decisions involving rewards on next actor’s social decisions involving rewards (all *p* > 0.05). Finally, we also scored the previous blocks using only the number of a given outcomes delivery and performed similar analysis. We found that both the numbers of airpuffs delivered to nobody and to the actor by the partner were significant predictors of the actor’s social decisions involving airpuff deliveries (Figure 2D, Number of airpuffs delivered to nobody: GLME, *R*^2^ = 0.57, *F* = 21.3, *p* < 0.001; Number of airpuffs delivered to the partner: GLME, *R*^2^ = 0.46, *F* = 10.3, *p* = 0.006). In other words, the number of airpuffs received and also avoided by the actor due to its partner decisions in the previous block influenced his own social decision in the next block. Similar analysis using the number of the outcomes from other types of decision was not significantly predicting the actors’ social decisions (Number of juice delivered to the partner: GLME, *R*^2^ = 0, *F* = 0.09, *p* = 0.76; number of juice delivered to nobody: GLME, *R*^2^ = 0.15, *F* = 1, *p* = 0.32; number of airpuff delivered to the actor by the actor: GLME, *R*^2^ = 0.13, *F* = 2.47, *p* = 0.14; number of airpuff delivered to nobody by the actor: GLME, *R*^2^ = 0, *F* = 0, *p* = 0.94).

## Discussion

Our results show that during dynamic exchanges, macaques’ social decisions can be influenced by the prior social decisions of a peer. In order to control for a potential non-social confound, we have assessed the influence of a prior self-induced airpuff on a future social decision and found no effect. This ruled out trivial explanations of our results implying that receiving an airpuff increased the monkey’s arousal, which in turn would have negatively affected its prosocial motivation or its general ability to make decisions. This suggests that witnessing the other monkey’s social decisions is necessary to induce the observed social decision modulations. Other alternative interpretations of our results can be considered. For instance, one could argue that the observed reciprocity was due to a mirroring of the partner’s previous actions (Suchak and de Waal, 2012). However, the actions themselves (i.e., pointing gesture toward an icon on a touch screen) were the same for all choices and the visual cues associated with each outcome were specific for each monkey and each type of decisions. Hence, advocating for such form of reciprocity would imply that the animals were not imitating the mere gesture of the partner (Nagasaka et al., 2013) or the choice of a specific visual stimulus but the action’s intended effect. Such line of interpretation would somehow relate our results to a social form of cognitive imitation (Subiaul et al., 2004). However, the fact that the observed effect persisted in time and the absence of reciprocity for the delivery of juice reward rules out the idea that our results can be solely explained by a purely reflective mechanism. Our relatively modest sample size is somehow compensated by our well controlled experimental design and the large number of trials in this dataset. Our data analysis thus reliably support the idea that the observed decisional bias is explained by a non-reflexive use of others’ behaviors to guide choices, as observed by other neuroscience studies in laboratory settings (Yoshida et al., 2011; Haroush and Williams, 2015). Ethological reports have shown that macaques can reciprocate positive behavior, such as social grooming (Schino, 2007) and can exhibit revenge-like behavior (Aureli et al., 1992; Silk, 1992). Our results could be thus interpreted as a proof of macaques following a tit-for-tat strategy during experimental social exchanges. It should be noted that, on average, the monkeys were prosocial as they significantly preferred to refrain from causing harm to their partners (for more details, see Ballesta and Duhamel, 2015). Therefore, tit-for-tat could not be a dominant strategy in the present case, as it should have led to an escalation of retaliatory actions and, in the long run, to a tendency toward antisocial decision-making. The use of retaliation was thus likely balanced by other cognitive processes such as the desire to preserve other’s welfare or social bonds (de Waal and Suchak, 2010; Yamamoto and Takimoto, 2012; Ballesta and Duhamel, 2015). Monkeys, therefore, might use retaliation parsimoniously which is consistent with comparable studies involving human subjects (Fitz et al., 1979, 1983). Additionally, tit-for-tat strategy could be interpreted differently as meaning that the monkey recognized its partner’s active airpuff withholding and thus increased it prosocial tendency as proof of gratefulness. At first, it seems easier to recognize the social motivation of others on the basis of the actual consequences of their action toward self (here, the delivery of an airpuff to the other monkey), compare to the absence of consequences (the delivery of an airpuff in an empty space). However, our results show that the number of airpuff delivery and avoidance are both sufficient to measure contingencies between social exchanges. This suggests that the observed tit-for-tat strategy may not only be motivated by retaliation after receiving an airpuff but also by the gratefulness of not having received one. This unexpected display of gratitude in a despotic non-human primate species calls for further investigations as this higher-order cognitive ability seems crucial to establish prosociality in humans (Ma et al., 2017). In fact, retaliation and gratitude are likely to depend on evolutionary rooted social cognition, as preverbal infants can identify the nature of the social motivation of individuals during the observation of social interactions (Kuhlmeier et al., 2003; Hamlin et al., 2007).

Monkeys can perform social exchange in different currencies (e.g., a female grooming a mother to gain access to her infant; Ventura et al., 2006; Carne et al., 2011; Borgeaud and Bshary, 2015). In our study, we did not find any evidence of such abilities to transform a social exchange into another form of social exchange. This negative result could be simply explained by the fact that our macaques did not reciprocate juice reward delivery. Recent ethological report shows the absence of short term contingencies between grooming and food tolerance in a different species of macaques (Molesti and Majolo, 2015) and in bonobos (Goldstone et al., 2016). This is somehow consistent with our results and underline the singularity of food sharing behaviors (Watson and Caldwell, 2009). It is thus legitimate to challenge the relevancy of food allocation tasks to study primates’ social behaviors (Watson and Caldwell, 2009; Jaeggi et al., 2013; Carter, 2014). It is unclear whether macaques can conceive the agency in a transfer of ownership, and whether this represents a relevant social cue. In macaques, the tolerance for co-feeding is unlikely to be bi-directional as it mainly occurs between the mother and her infant, or between a potent and a more subordinate individual (Ventura et al., 2006; Massen et al., 2010; Jaeggi and Van Schaik, 2011; Dubuc et al., 2012). In macaque despotic societies dominant individuals naturally have a “right of preemption” on the subordinates’ goods which likely made reciprocal exchanges of food irrelevant. Future studies should take these observations into consideration, especially when performances in juice reward allocation tasks are taken as a proxy of macaque’s social motivation in neuroscience (Chang et al., 2011; Azzi et al., 2012). To overcome these methodological and conceptual challenges and study exchanges of positive acts under experimental condition in primates, the use of grooming by the experimenters (or by a dedicated device) might be considered in further investigations (Taira and Rolls, 1996; Grandi and Ishida, 2015). To conclude, by using both positive and negative outcomes, this study has clarified the apparent dichotomy within the scientific literature of reciprocity in non-human primates. We found contingencies in the social exchange of negative acts in macaques and show that macaques can use tit-for-tat strategies and gratitude in their social exchanges which represent a new insight into the nature of non-human primates’ social cognition. These results lay the foundation for the investigations of the cognitive and neural basis of reciprocity in exchange of negative acts in non-human primates.

## Data Availability Statement

The raw data supporting the conclusions of this manuscript will be made available by the authors, without undue reservation, to any qualified researcher.

## Ethics Statement

The animal study was reviewed and approved by Department of Veterinary Services, Health & Protection of Animals, permit number 69-029-0401 Biology Department of the University Claude Bernard Lyon 1.

## Author Contributions

SB, GR and J-RD performed analyses, discussed the results and contributed to the text of the manuscript. SB performed the experiment. All authors reviewed the manuscript.

## Conflict of Interest

The authors declare that the research was conducted in the absence of any commercial or financial relationships that could be construed as a potential conflict of interest.
